# 

**Published:** 1908-06

**Authors:** 


Sixty-third Year.

VOL. LXIII. JUNE, 1908. NO. 11

### BUFFALO MEDICAL JOURNAL

LIBRARY
OFFICE

Established 1845 by AUSTIN FLINT M. D.

LIBRARY
Indexed

EDITOR:

WILLIAM WARREN POTTER, M. D.

ASSISTANT EDITORS:

WILLIAM C. KRAUSS, M. D. NELSON W. WILSON, M. D.

ASSOCIATE EDITORS:

JAMES WRIGHT PUTNAM, M. D. ERNEST WENDE, M. D.
JOHN PARMENTER, M. D. JOHN A. MILLER, PH. D.
MAUD J. FRYE, M. D.

